# Methodology used to investigate the energy savings of opaque ventilated façades in residential buildings in Brazil

**DOI:** 10.1016/j.mex.2021.101227

**Published:** 2021-01-11

**Authors:** Ana Carolina Fernandes Maciel, Michele Tereza Carvalho

**Affiliations:** Universidade Federal de Uberlândia, Universidade de Brasília, Brazil

**Keywords:** Opaque ventilated facades, Operational energy, Energy efficiency simulation

## Abstract

Opaque ventilated facades have as it main advantage reduce cooling thermal loads, thus reducing the energy consumed by artificial systems. This paper presents the method used by Maciel and Carvalho (2019) [1], that investigated the energy benefit of opaque ventilated façades compared to cladding façades in multi-floor residential buildings in Brazil. The authors divided the methodology in four parts: ensure the gap of investigation through a systematic mapping of literature (SML), verify the best software´s BIM and BES, in terms of interoperability, performed several simulations during a hole year for 09 different climate regions, and validated the collected data with a statistic tool. In the SML few studies were found on the topic discussed, and only two performed comparisons between different climates, both used computer simulation. The bibliographic revision about interoperability showed that the most comprehensive BES software´s to use with a BIM model were IES-VE and GBS, being chosen the GBS. All configuration to the simulation were made following instructions of international regulations. The collected data was validated by Tukey test. Although the steps of the methodology were not original, the authors certified along the process that the choices made were the most efficient and valid ones.

• With this methodology, it was possible to gain great agility in data being generated, which enables a broad sample and a several possibilities of analyses.

• The statistical processing of data enables trust in the results found with the computer simulations.

## Graphical abstract

**Figure:**
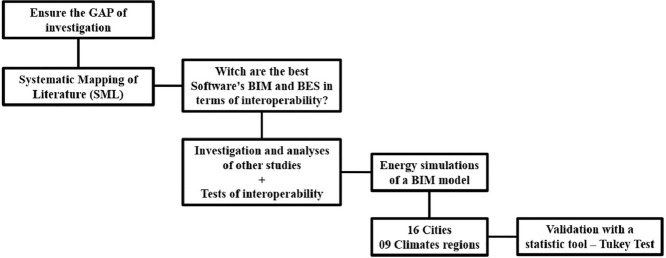

Specifications table

**Table:** 

Subject Area	*• Energy*
More specific subject area:	Energy efficiency simulation
Method name:	*Computational simulation and statistical analysis*
Name and reference of original method	*This article was based on Ryu and Park**[2]**who studied the energy simulation process using BIM and described the modeling methodology for the ASHRAE 90.1 based on energy analysis parameters for BIM software, Autodesk Revit, error checking and energy analysis with Trace700. This paper aims to analyze the method used by Maciel and Carvalho (2019)**[1]**. It was decided to use the BES software for energy analysis, based on Sanhudo et al.**[3]**that reviewed the literature on BIM studies for retrofits, where they verified among other items the best integrations between software and energy analysis methods, recommending software that export the data as gbMXL with integration between them.*
Resource availability	

## Method details

It´s known that energy consumption is a concern around the globe and the civil construction sector is one of the most responsible for this consumption [2,^4^,5], and is a challenge required to developed and developing countries. The performance of building envelopes plays a fundamental role in the impact and needs of building heating and cooling [6].

The research of the authors [1] investigated the energy savings of opaque ventilated façades compared to cladding façades in multi-floor residential buildings in Brazil located in nine climate zones, according to Köeppen-Geiger classification. This type of facades was chosen because it´s a relatively new industrialized element to facades at the country. Also, it´s a system that under the solar radiation effect, has energy performance better than conventional facades [6], having as the main characteristic the ability to reduce cooling loads [7].

This paper aims to analyze the method used by Maciel and Carvalho (2019) [1]. The methodology used was divide in four main parts: 1) ensure the gap of investigation through a systematic mapping of literature; 2) verify the existing market options of software´s and the better options in terms of interoperability between BIM and BES software´s and performed the interoperability tests needed; 3) performed several simulation during a hole year for 16 different cities and 4) validated the data with a statistic tool.

## Systematic mapping of literature

Brazil is a country that has a vast territory and very few studies towards the performance of facades, in special covering a wide range of cities and climate zones. Therefore, to understand better the position of the proposed theme in the scientific community in the world, the authors conducted a systematic literature mapping (SLM).

With this part of the method the authors were able to conclude that although there is a considerably large amount of studies and journal papers related to “ventilated envelopes”, most of them are focused on double skin façades, building integrated photovoltaic, solar chimney, trombe, and solar walls, or façade solar collectors. However, less attention has been given to opaque ventilated façades (OVFs) [8].

The systematic literature mapping used the Scopus and web of science databases to search national and international articles, in Portuguese and English between 2010 and 2018. The Boolean operator AND was used with the following sets of keywords: operational energy and ventilated façade, energy consumption and ventilated façade, thermal performance and ventilated façade, energy efficiency and ventilated façade, operational energy and BIM, ventilated façade and BIM, ventilated façade and energy simulation, as well as ventilated façade and climate zone [1].

The applied filters allowed 613 articles. After reading the titles, all those that did not deal with the opaque ventilated façade (double glass façade, PCM façade, photovoltaic, etc.) were excluded from the study. The remaining 84 titles were reduced to 56 articles, after excluding the titles in duplicate and, after reading the abstracts to 25 articles, which were read in full and classified [1].

There is a considerably large amount of studies and journal papers related to “ventilated envelopes” especially focused on double skin façades, building integrated photovoltaic, solar chimney, trombe, and solar walls, or façade solar collectors. However, less attention has been given to opaque ventilated façades (OVFs). There are few studies on opaque ventilated façades, about two to three studies published per year, in Europe, especially Spain and Italy [1].

Only two studies from the analyzed 25 consider more than one climate zone and most do not mention the climate classification used. Therefore, a globally recognized classification, the Köeppen-Geiger was used in this study. Most studies compare opaque ventilated façades to sealed ventilated façades, emphasizing the need for a study comparing the benefits of the air chamber of ventilated façades to cladding façades, without the air chamber [1].

Of the 25 papers, seven were experimental studies using test-cells with stable climatic conditions; eight were simulations with CFD modules of a specific cell or facade, analyzed only the thermal behavior of the airflows inside the chambers, without comparing with another type of façade; six were numerical studies while only three addressed energy analyses using BIM, and some of the studies duplicated numerical, experimental and energetic simulations. And, most of the studies consider a single season of the year [1].

Regarding this part of the methodology, the authors made a wide quest about the theme and were able to validate the knowledge gap, showing that studies about this theme are still relevant. This procedure is very common and necessary in this kind of research.

## Interoperability between BIM and BES software's

To decided what software would be used to the energy simulations, a search for articles of interoperability were performed and the possibility of use were analyzed.

Using BIM in building performance analysis can facilitate a more accurate and efficient analysis process. However, in order to perform a successful BIM-based building performance analysis, it is necessary to secure the interoperability between a BIM based architectural model and analysis programs [9].

According to Pan, Qin and Zhao (2017) [10], the best approach to energy modeling of buildings is the one that integrates the design process with the energy modeling process. Use building information modeling (BIM) tools, such as Revit, to create building geometry and apply all energy analysis settings/configurations. Subsequently, this information can be exported as a gbXML file that is recognized by the energy simulation software efficiently.

Ruy and Park [2] state that BIM data can be integrated with energy performance simulation software such as Green Building Studio (GBS), Ecotect and Project Vasari, and this integration makes them powerful plugging tools for designing a high-performance energy building.

Based on the authors and on the knowledge about some software's, Maciel and Carvalho search for more literature that could certified their choices. They had already worked with Design Builder and Ecotect in the past and had familiarity with Revit, so this connection would be favorable.

BES tools require input of building surface information, material properties, system description, and weather data to compute the dynamic heat transfer for a specified time period. In current practice, many architects develop the building design data in the form of digital models such as BIM models, which have the building geometry and material information necessary for a thermal simulation. However, in many cases this information cannot be directly exchanged between BIM and BES tools. This can be a significant problem that causes delay in the process of studying the building performance in the early design stages where major design decisions are made toward designing sustainable and high-performance buildings [11].

Some existing simulation tools were modified for data exchange capabilities between BIM and building energy simulation (BES) through standard data schemas such as IFC and gbXML, which contain building geometry information and other information of internal loads, occupancy, zone assignments, system configuration, and utilization schedules [12].

Dong et al. [13] affirmed that IFC and gbXML schemas are supported by BIM tools including Revit, Bentley, and ArchiCAD, as well as energy simulation tools such as Green Building Studio, Ecotect, Hevacomp, eQUEST, HAP, and IESVE.

Moon et al [9] evaluate the interoperability between BIM based architectutal model and building performance analysis programs. The study scope was limited to evaluating the interoperability using a BIM model created from Revit 2011 with EnergyPlus 5.0, eQuest 3.64, Ecotect 2011(discontinued in 2015) and IES-VE 6.1. The study also used Green Building Studio (GBS) as an interface to Energy Plus and eQuest to import a gbXML file indirectly, since this software´s were unable to import a gbXML file directly.

All of the four analysis programs were able to be imported building geometry information including surfaces (floor, wall, roof), opening (window, door) from the BIM based model. However, EnergyPlus showed some problems in converting building geometry such as the location of the openings. eQUEST (with GBS) showed the most compatible data exchange ability among four energy analysis programs [9].

Kim et al. [14] points that, since 1996, more than four hundred software tools for building energy simulation have been listed in the “Building Energy Software Tools Directory” provided by U.S.DOE (2013) [15]. Among the tools, a few are dominantly used in industry, as DesignBuilder, DOE-2, eQuest, Ecotect, Energy-10, EnergyPlus, Green Building Studio, HEED, and IESVE.

DesignBuilder can import three-dimensional geometry information through gbXML and two dimensional building footprints through DXF. OpenGL solid modeler of DesignBuilder visualizes building façade design and solar studies [15].

As the literature points, Design Builder wouldn´t be the best one, since his interoperability with BIM has not been proved to be efficient. To an energy analysis several data besides geometry information is need, as internal loads, occupancy, zone assignments, system configuration, and schedules. Also, Ecotect is discontinued since 2015, so it couldn´t be chosen as well.

Some simulation tools can be plugged into BIM authoring tools as add-ins such as IESVE and Green Building Studio, which allow energy simulation within the BIM environment. Green Building Studio is a web-based energy analysis environment based on DOE-2.2. Energy models are generated from Autodesk Revit models via certain manual preparation processes. Then, simulation is performed by a cloud service and the results are reported to users. IESVE has a Revit plug-in that can generate an energy model based on gbXML in Revit but editing the exported energy model can be done in the IESVE interface [14].

BIM software has advantages in analyzing whole building energy performance. One advantage is that BIM software improves the usability of the whole building energy calculation by using standard processes and parameters [16]. BIM software follows a standard process to calculate whole building energy consumption based on various parameters such as building use patterns, building shape, materials, and weather conditions [17]. These are generally default parameters retrieved from an external database where information has already been collected from surveys of standard practices [18].

Some studies indicate that the family of BIM software systems should holistically cover the various aspects of buildings, and tools for sustainability analysis, which is further illustrated in Azhar and Brown [19] and Azhar et al. [20], which found that there are three commonly used BIM-based sustainability analyses software, namely Autodesk Ecotect™ (which has been ended since 2015), Autodesk Green Building Studio (GBS)™, and IESVE. In accordance with those, Lu et al [18] selects and reviews 12 BIM analysis tools from the BIM Tools Matrix [21]. Regarding the functionality, these 12 types of software can contribute to the sustainability analysis of green buildings in six aspects, including energy consumption, carbon emissions, natural ventilation, solar radiation and lighting, acoustics, and water usage. A summary of the software's to energy analysis from these 12 BIM tools is display at Table 1.

**Table 1:** Popular types of BIM software and their functions used for green building analyses [adapted from Lu et al [18].

BIM Software	Green analysesa					Usersb	Usersc
	E	CE	NV	SD	W		
Green Building Studio	√	√	√	√	√	A/D	De/OM
IES-VE	√	√	√	√	√	A/D/E/O	De
Bentley Hevacomp	√	√	√			D/E/C	De
AECOsim	√	√		√		E/C/D	De
Energy Plus	√	√		√	√	E/A	De
HEED	√	√				O/A/D/C	De
Design Builder Simulation	√	√	√	√		C/E/A	De
eQUEST	√		√	√		A/E/C	De/C/OM
DOE2	√		√	√		A/E/C/G	De
TRNSYS	√		√	√		A/E	De

^a^E for energy, CE for carbon emissions, NV for natural ventilation, SD for solar and day lighting, W for water.

^b^A for architects, D for designers, E for engineers, O for owner, C for consultants, G for government.

^c^De for design, C for construction, OM for operation and maintenance.

Some BIM software provides broad and comprehensive sustainability analyses, such as Green Building Studio (GBS). GBS is mainly used in the design phase, but it also serves various end users, including architects, designers and engineers, as well as other project participants whose work can benefit from using BIM applications, such as consultants, owners, and contractors [22]. Another example is Integrated Environmental Solutions® Virtual Environment (IES-VE), which visualizes sustainability issues in project delivery and helps owners determine the most optimized green-design solutions [23].

As presented, there are several different tools to modeling and energy analyses. Based on the studied authors the most comprehensive software´s are GBS and IES-VE, since DBS has too many interoperability issues and Ecotect is discontinued. IES-VE and GBS can be plugged into BIM authoring tools. GBS can be used at design and operational and maintenance and IES-VE just in design. Also, IES-VE as DBS needs some edition at the BES software after exportation, what can cause interoperability problems. As GBS is from the same developer than Ecotect and Revit, and the author already had knowledge of those system, so Revit and GBS software's were chosen to the research.

The authors did make some tests of interoperability between Revit and DBS and confirmed what already had read about it. DBS imports just geometry, and complex geometry are simplifying in the process, and much data are lost, showing that at the present time of the research (2018) that Revit and DBS doesn´t present the interoperability need. After that, several simulations were made with Revit and GBS in order to verify interoperability issues and the results were promising. The test was made with a single room with door and window, with all the configurations used to the research. Also, the weather data of GBS was compared to weather data of the Brazil government webpage to validate temperatures, precipitation, humidity, etc.

The annual simulations considered the energy consumption by the artificial air conditioning and lighting systems. BIM Autodesk Revit 2018 software was used for modeling the building and configurations, and the cloud service, green building studio (GBS) for energy simulation, which has its database linked to DOE 2.2, and Energy Plus. Weather data originated from Revit virtual climate stations, which provide year-round data based on a 30-year average climate data in formats such as the Meteorological Year (TMY2).

The GBS choice provide time reducing, facilitated the simulation process and limited the possible errors, since is a web-based service, connected directly to Revit, being not necessary to export/import files, which is performed automatically by Revit.

## Simulations

Before beginning the simulations two principal models were built on Revit® software: one with granite cladding façade (GCF) and the other one with granite ventilated façade (GVF).

The building architectural design was developed by the authors and based on Brazilian residential standards and the most commonly used construction pattern in the country, which according to CBIC (2017) [24] is the R8-N, a normal residential eight-story residential building. The National Standard Basic Unit Cost Model (CUB) R8-N [25] requires Garage, Pilotis and Eight floors, being:

Pilotis with stairs, elevators, entrance hall, ballroom, small kitchen, two bathrooms, central gas and guardhouse.

Typical floor with circulation hall, stairs, elevators and four apartments.

Each apartment with three bedrooms, including a master with en-suite bathroom, living/dining room, social bathroom, kitchen, service area with bathroom and balcony

**Fig. 1:**
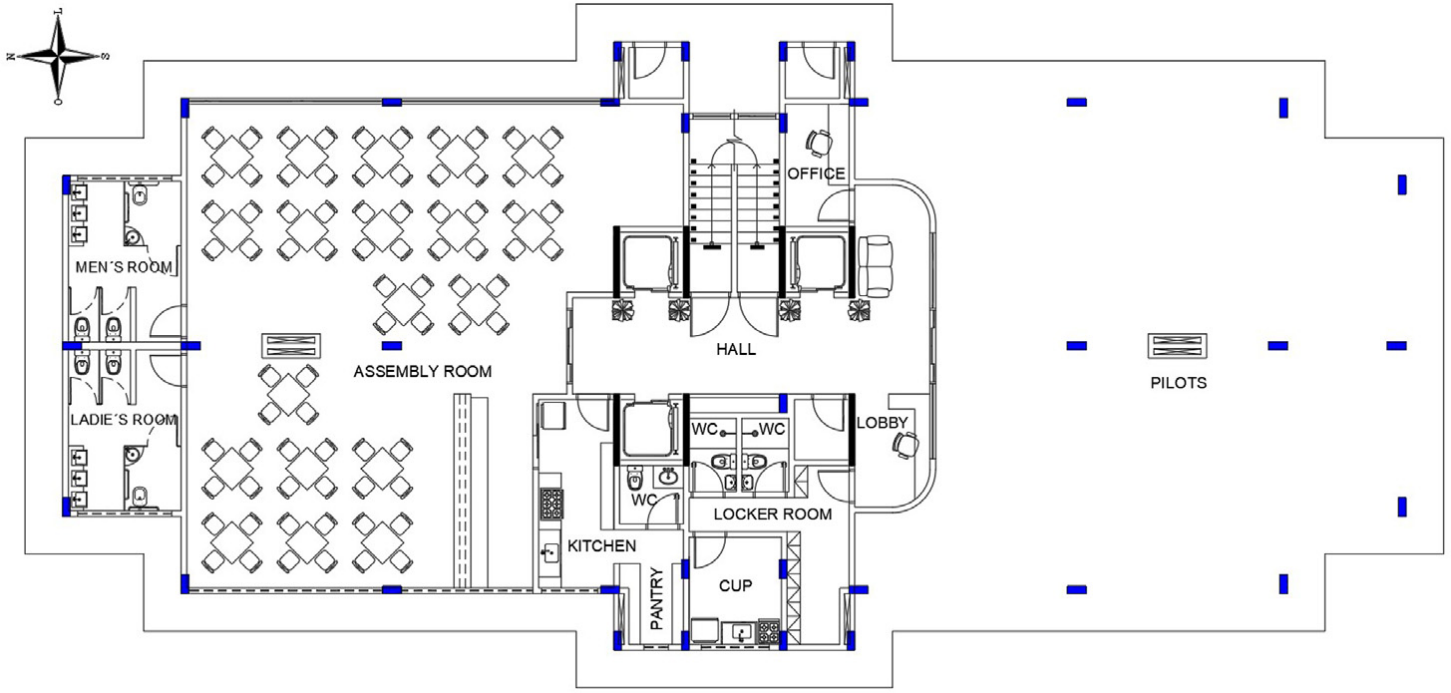
Blueprint of the pilotis.

**Fig. 2:**
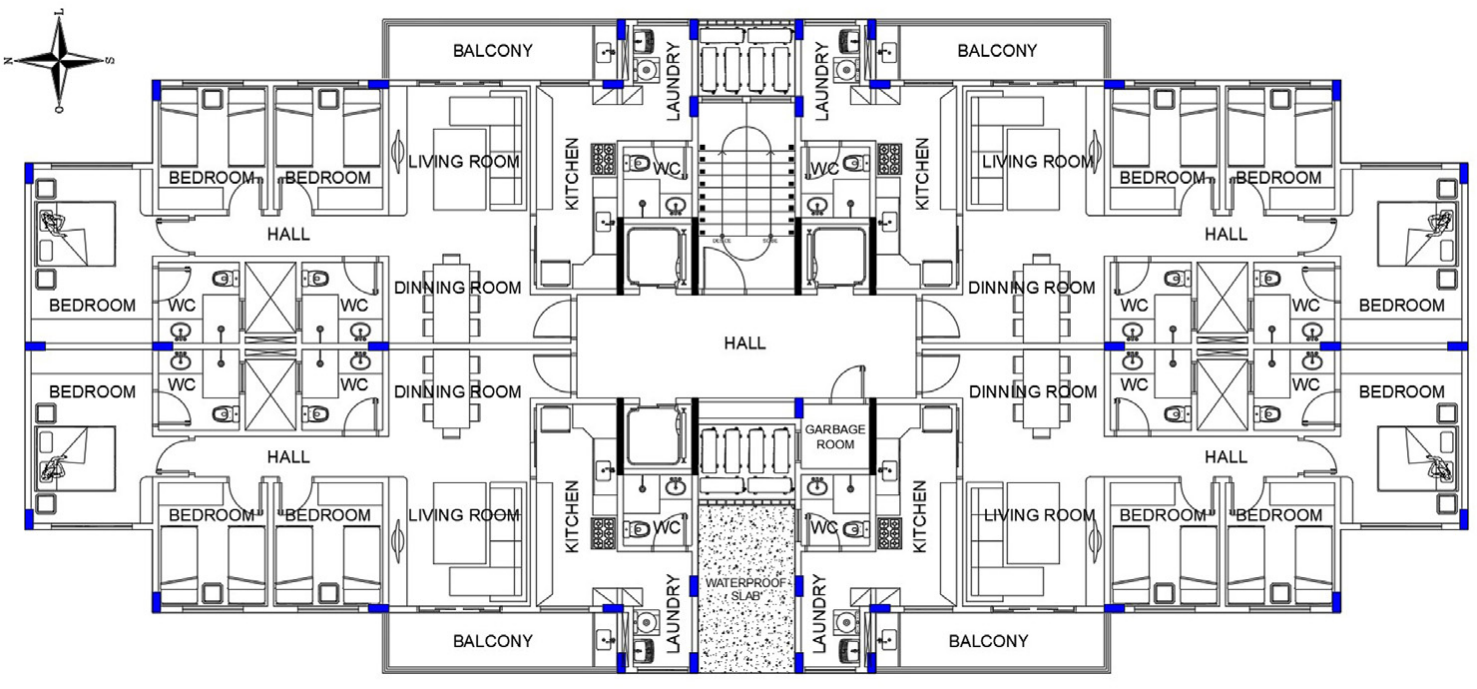
Blueprint of a typical floor.

**Fig. 3:**
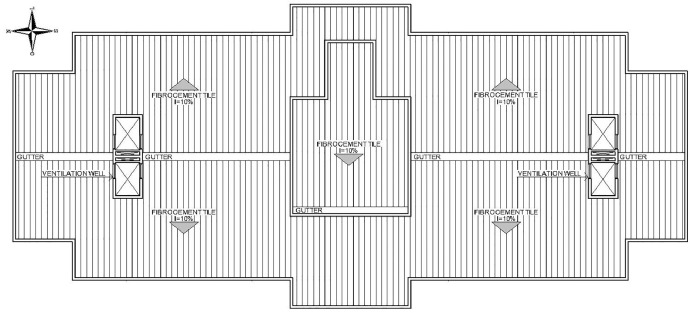
Blueprint of the roof.

Fig. 1 shows the floor plan of the pilotis with a ​​291 m² floor area while Fig. 2 shows the typical floor plan with 448.30m² area and, Fig. 3, the floor plan of the roof, which is not specified in the Standard Project of CUB, thus being designed for the best cost benefit for this type of building.

**Fig. 4:**
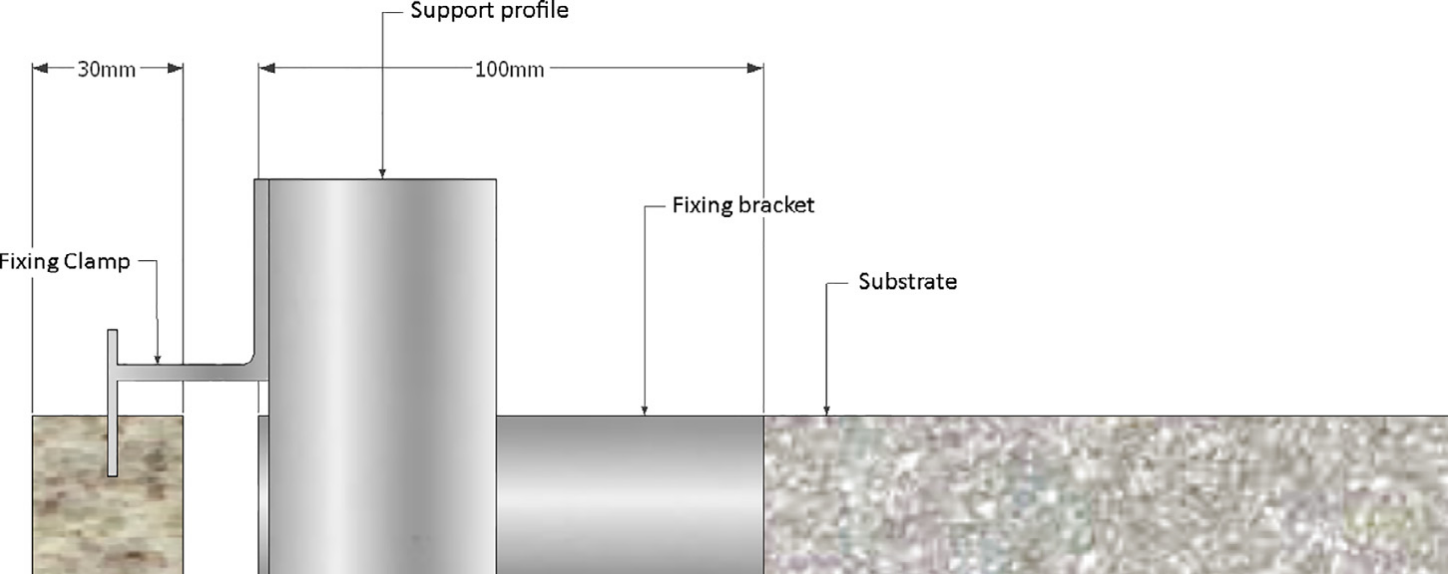
Schematics of a GVF transversal section (in mm).

**Fig. 5:**
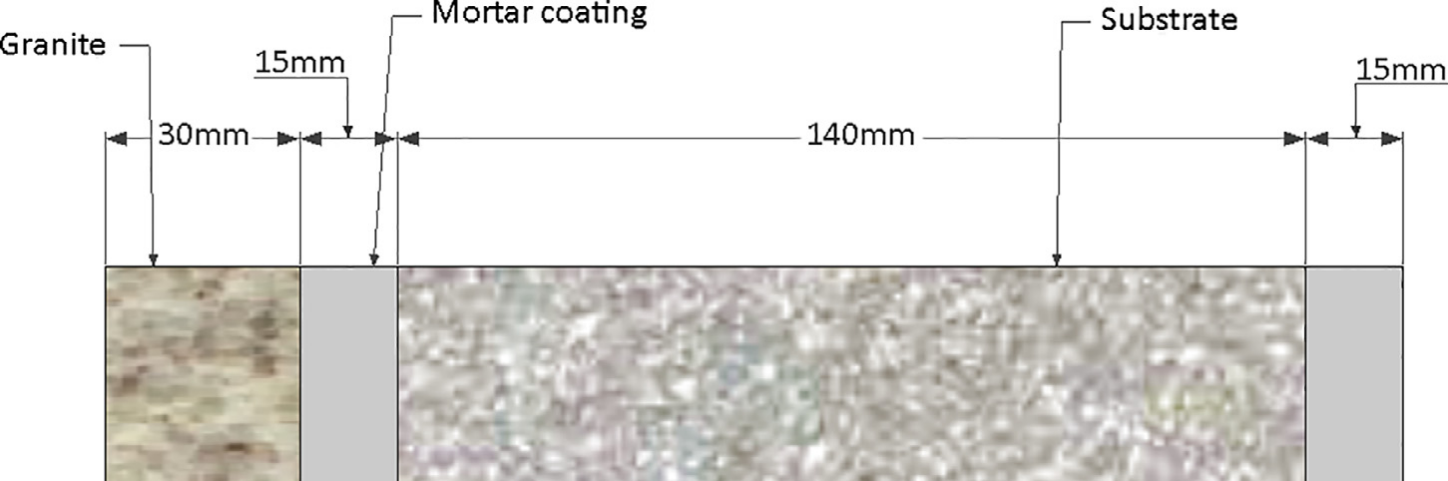
Schematics of a GCF transversal section (in mm).

The Granite Ventilated Facade (GVF) had a 100mm thick air chamber and a 30mm granite plate (Fig. 4). The Granite Cladding Facade (GCF) was described according to Junginger [26] as: base or substrate, roughcast, regularization layer, fixing layer and coating, which is 30 mm thick (Fig. 5). The standard thickness is 20 mm and 30 mm for granite cladding and ventilated façades respectively, however, to avoid interference of coating thickness in the results, the thickness of the ventilated façades was adopted for both cases (30 mm).

**Fig. 6:**
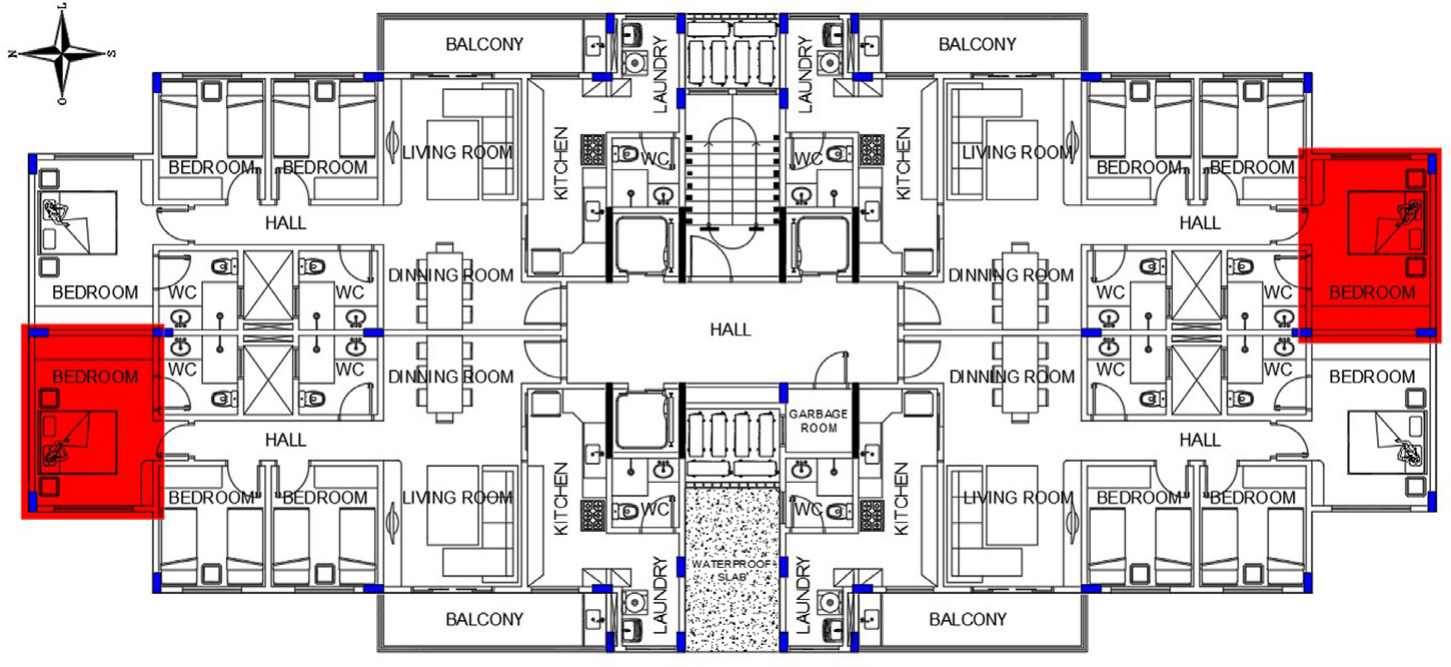
Solar orientation for EHM.

The solar orientation for the façades was defined in the most unfavorable configuration possible [8] as it can be seen at Fig. 6. The Brazilian performance standard ABNT NBR 15575-1 [27] recommends that when analyzing the thermal performance of residential buildings with multiple floors, the building solar orientation should be positioned as to evaluate the most critical condition unit from the thermal viewpoint:

• Summer: dormitory or room window facing west and another exposed wall facing north;
• Winter: dormitory or living room window facing south and another exposed wall facing east.

The north cardinal point, according to Fig. 5, meets the most critical condition from the thermal viewpoint in the summer and partly in the winter. The winter condition was not applied completely because the building has opposite apartments, leaving the bedroom window to the east and another wall to the south being, therefore, the inverse of the bedroom that meets the critical summer condition*.*

After the models were ready, several configurations were necessary. Climate classification were analyzed by three classifications being ABNT NBR 15220 [28], ASHRAE 90.1 [29] and Köeppen-Geiger classification [30], after that they decided to base the study on the most comprehensive of the three (Köeppen-Geiger). Brazil is divided into nine climate zones according to the adopted classification and this study investigated 16 Brazilian cities in all nine-climate zones as shown in Table 2.

**Table 2:** Cities and Climate Zones analyzed in this study.

State	City	Climate region
Pará	Belém	Af - Equatorial climate fully humid
Amazonas	Manaus	Af - Equatorial climate fully humid
Bahia	Salvador	Af - Equatorial climate fully humid
Alagoas	Maceió	Am - Equatorial climate monsoonal
Sergipe	Aracaju	As - Equatorial climate with dry summers
Maranhão	São Luis	As - Equatorial climate with dry summers
Distrito Federal	Brasília	As - Equatorial climate with dry summers
Mato Grosso	Cuiabá	Aw - Equatorial climate with dry winters
Mato Grosso do Sul	Campo Grande	Aw - Equatorial climate with dry winters
Alagoas	Água Branca	BSh - Arid Climate hot steppe
Rio Grande do Sul	Porto Alegre	Cfa - Temperate climate, fully humid with hot summers
São Paulo	São Paulo	Cfa - Temperate climate, fully humid with hot summers
Paraná	Curitiba	Cfb - Temperate climate, fully humid with warm summers
Minas Gerais	Belo Horizonte	Cwa - Temperate climate with dry winters and hot summers
Rio de Janeiro	Nova Friburgo	Cwb - Temperate climate with dry winters and warm summers

The data for configuration the heating and cooling systems follows the ISO 17772-1 [31], considering for energy consumption analysis, with a minimum temperature of 20°C and maximum temperature of 26°C, for level II – medium^1^.

For residential buildings, ASHRAE 90.1 [29] recommends using a single air conditioner per room - window or split system for cooling and/or heating. The "RESIDENTIAL 17 SEER/9.6 HSPF SPLIT HP <5.5 TON" equipment used was among the options provided by the software. The HVAC zoning system was used to activate it for bedrooms and living rooms. The Annex G3 of the standard ASHRAE 90.1 [29] states that HVAC systems must be activated/deactivated according to the occupancy schedule but does not provide it. This schedule varies according to the building type and is, usually, already predefined in the software. The ISO 17772-1 standard [31] suggests a few schedules and the multifamily housing schedules were used in this research (Tables 3 and 4).

**Table 3:** Residential apartment: Parameters and Setpoints [adapted from 31].

	Parameter	Value	Unit
Operation time	START, time of the day	0	Hour
	END, time of the day	24	Hour
	Intervals	0	Hour
	Days/week	7	Days
	Hour/day	24	Hour
	Hour/day	8760	Hour
Internal thermal gain	Occupants	28.3	m²/person
	Occupants (total)	4.2	W/m²
	Occupants (dry)	2.8	W/m²
	Equipment	3	W/m²
	Lighting (rooms/bedrooms)*	8.4/6.57	W/m²
	Moisture production	2.12	g/(m².h)
	CO2 production	0.66	l/(m².h)
Setpoints	TO minimum	20	°C
	TO maximum	26	°C
	Ventilation rate	0.5	l/(s.m²)
	Ventilation rate for CO2 emission	0.28	l/(s.m²)
	Maximum CO2 concentration	500	Ppm
	Minimum relative humidity	25	%
	Maximum relative humidity	60	%
	Lighting in work spaces	0	lx

⁎ The lighting and occupancy parameters are used to simulate the internal heat gain, which impacts the energy consumption by the HVAC systems.

**Table 4:** Usage schedule [adapted from 31].

Energy calculation						
	Weekdays			Weekends		
Time	Occupants	Equipment	Lighting	Occupants	Equipment	Lighting
1	1	0.5	0	1	0.5	0
2	1	0.5	0	1	0.5	0
3	1	0.5	0	1	0.5	0
4	1	0.5	0	1	0.5	0
5	1	0.5	0	1	0.5	0
6	1	0.5	0	1	0.5	0
7	0.5	0.5	0.15	0.8	0.5	0.15
8	0.5	0.7	0.15	0.8	0.7	0.15
9	0.5	0.7	0.15	0.8	0.7	0.15
10	0.1	0.5	0.15	0.8	0.5	0.15
11	0.1	0.5	0.05	0.8	0.5	0.05
12	0.1	0.6	0.05	0.8	0.6	0.05
13	0.1	0.6	0.05	0.8	0.6	0.05
14	0.2	0.6	0.05	0.8	0.6	0.05
15	0.2	0.6	0.05	0.8	0.6	0.05
16	0.2	0.5	0.05	0.8	0.5	0.05
17	0.5	0.5	0.2	0.8	0.5	0.2
18	0.5	0.7	0.2	0.8	0.7	0.2
19	0.5	0.7	0.2	0.8	0.7	0.2
20	0.8	0.8	0.2	0.8	0.8	0.2
21	0.8	0.8	0.2	0.8	0.8	0.2
22	0.8	0.8	0.2	0.8	0.8	0.2
23	1.0	0.6	0.15	1.0	0.6	0.15
24	1.0	0.6	0.15	1.0	0.6	0.15

The locations of weather stations followed the instruction of ASHRAE 90-1 [29], being the airport meteorological station found through the Internet Mapping Service of Revit® software. The lighting configuration followed the recommendation of the same standard: 8.40 W/m2 for the living rooms and 6.57 W/m2 for the bedrooms.

The used materials and components are described in Table 5, considering its location of use, thickness and properties of thermal conductivity (λ), specific heat (c), and density. All materials and systems of the models were created and configured in the software, while the properties of the materials are from the database itself, following the standard ASHRAE 90.1 [29]. The configuration of the systems was based on data from the Brazilian construction market. The U and CT data are calculated by the software.

**Table 5:** Description of the materials used in the simulations.

Local	Material	Thickness (mm)	λ (W/m.k)	c (kJ/kg.k)	Density (kg/m³)
External walls (GCF)	Granite	30	3.49	0.79	2880
	Plaster	15	0.72	0.84	1860
	Concrete	20	1.0460	0.657	2300
	Air chamber	100	0.0250	1.0035	1.20
	Concrete	20	1.0460	0.657	2300
External walls (GVF)	Concrete	20	1.0460	0.657	2300
	Air Chamber	100	0.0250	1.0035	1.20
	Concrete	20	1.0460	0.657	2300
Glass Frames	Anodized aluminum trim	–	230	0.897	2700
	Common colorless glass (6mm)	6	1.10	0.84	2480

Obs.: The concrete block was configurated as Concrete + Air chamber + concrete, as it is show above.

The Revit® software automatically calculates the values ​​of U and R, based on the thermal properties of the materials presented on Table 6. These values ​​are shown in Table 5. Externally to the external walls of the building to the GVF model, a curtain wall with openings at the top and bottom of the façade was installed. The gap between the external wall and curtain wall was 100mm.

**Table 6:** Values of U and R to External walls.

Local	Material	Thickness (mm)	U (W/(m².K))	R ((m².K)/W)	Thermal mass (kJ/K)
External walls (GCF)	Granite + Plaster + Concrete block	185	0,2458	4,0677	14,15
External walls (GVF)	Concrete block	140	0,2476	4,0382	5,63
Curtain wall	Granite	30	2,8746	0,3479	—

The simulations generated an immense variety of data, which had to be validated (Title 5) and analyzed for the research results. To validate the data, it was separated for cities and for consumed energy of heating and cooling systems. The achieved results agree with the expected from the authors, once the behave of climate regions are similar to other studies realized in Europe. Some results showed necessity of wider climate region classification, especially to countries with so many variables.

## Validating data

The results of the simulations were charted and underwent statistical treatment using Tukey test to compare the averages according to Levin [32]. The averages were compared two by two to determine significant differences for a pre-determined level. If in a set there are five averages, for example, the tool allows comparing two by two to detect whether they are statistically different in relation to the stipulated confidence level.

The Tukey test is based on the Honestly Significant Difference (HSD) value, which is given by Eq. (1):

(1) $$HSD=q_{\alpha \text{;}k\text{;}qk\left(n-1\right)}\cdot \sqrt{\frac{S_{Dentro}^{2}}{n}}$$

where $q_{\alpha ;k;k(n-1)}$ is the Tukey test amplitude with α significance level, with k treatments and n repetitions or observations, n is the number of months analyzed, and k(n-1) is the error (degrees of freedom). And Eq. (2) is the quadratic means of the variances within the k treatments [32].

(2) $$s_{Dentro}^{2}=\frac{s_{A}^{2}+s_{B}^{2}+s_{C}^{2}+\cdots +s_{k}^{2}}{k}$$

For the calculations, at 5% significance level (95% reliability), the values ​​closest to a specific set were used while every month values were used for some cities. The HSD value should be interpreted as being the highest value so that there is no difference between two considered averages, that is, if the difference between two averages is greater than HSD, then the averages are statistically different; if it is less than HSD, then the two averages are statistically equal at the determined significance level.

The Tukey Test was carried out for all cities in the two categories (heating and cooling). For the sake of simplicity and exemplification, Table 7 shows the parameters and the Tukey test summary to compare the heating averages whereas Table 8 shows the cooling results for the Granite Cladding Façade and Granite Ventilated Façade in Porto Alegre, RS. The heating average considered three months (May, June and September), whose values ​​are close and representative of the heating parameter. For Porto Alegre, the difference between the averages is 91.67, and smaller than the 831.61 HSD value; therefore, the averages are equal and must have the same code (a). In this case, the type of facade does not interfere with the energy consumed for heating.

**Table 7:** Tukey test, FCG x FVG Heating, Porto Alegre/RS.

Tukey test parameters			
n	3	qα;gl1;gl2	3,93
k	2	$S_{\text{Dentro}}^{2}$	134330,67
α	0.05	HSD	831.61
Heating	Average	Difference	Code
GCF	2976.60	225.00	a
GVF	2751.60		a

**Table 8:** Tukey test, Cooling, FCG x FVG, Porto Alegre/RS.

Tukey test parameters			

n	4	qα;gl1;gl2	3.46
k	2	$S_{\text{Dentro}}^{2}$	2282488.33
α	0.05	HSD	2613.67

Cooling	Average	Difference	Code
GCF	12009.00	2760.00	a
GVF	9249.00		b

The cooling variable considered the period from December to March (four months). In this case, the difference between the averages is 2760 and greater than the 2613.67 HSD value; therefore, the cooling averages are statistically different and must have different codes (a and b). In this case, the type of façade interferes with the energy consumed for cooling.

Simultaneously to the statistical treatment, the implantation costs were surveyed for Granite Cladding Facade and Granite Ventilated Facade with a structural system and punctual system, for non-coastal and coastal cities. The total costs of the facades for the studied building were determined and later divided by its square footage. This part of the methodology was not analyzed to this paper.

Although the adoption of statistic is not a new procedure, this stage of methodology it´s an important part of the study, because the authors needed to ensure the validation of the data collected.

## 6 Conclusions

This paper presents the method used by Maciel and Carvalho [1], that investigated the energy benefit of opaque ventilated façades compared to cladding façades in multi-floor residential buildings located in nine climate zones in Brazil. The following conclusions are made.

A Systematic mapping of literature were performed to secure the originality of the scope of the research. Few studies were found on the topic discussed, and the most of them analyzed the temperature behavior inside the facade chamber, in a single city. Only two research's performed comparisons between different climates, in 2014 in Europe and in 2019 in Brazil, both used computer simulation.

The authors search for the most comprehensive software, in terms of interoperability, thought several studies, searching from the most recent ones, that evaluate most recent versions of software´s, since developers improves these abilities at each version. This bibliographic revision showed that the most comprehensive BES software´s to use with a BIM model were IES-VE and GBS. IES-VE and GBS can be plugged into BIM authoring tools. GBS can be used at design and operational and maintenance and IES-VE just in design. IES-VE needs some edition at the BES software after exportation, what can cause interoperability problems. GBS is from the same developer than Ecotect and Revit, and the author already had knowledge of those system, so Revit and GBS software's were chosen to the research and the authors made some tests to improve the reliability of obtained data.

All configuration to the simulation were made following instructions of regulations as ASHRAE 90.1 [29], ISO 17772 [31] and NBR 15575 [27], which brought more reliability to the process. After all data were collect, it was charted and underwent statistical treatment using Tukey test to compare averages two by two to determine significant differences for a pre-determined level, using 5% significance level or 95% of reliability. The Tukey Test was carried out for all cities in the two categories (heating and cooling).

As the main contribution, this paper shows that with the methodology adopted by the authors [1], were possible to gain great agility in data being generated, which enables a broad sample and several possibilities of analyses. Also, the interoperability issues encountered at the process doesn´t interfere with general results. The authors certified along the process that the choices made were the most efficient and valid ones. Although the methodology steps individually were not original, together provides reliability to the study.

## Declaration of Competing Interests

The authors declare that they have no known competing financial interests or personal relationships that could have appeared to influence the work reported in this paper.

## Footnotes

1 The ISO 17772-1 [31] standard establishes different categories for temperature ranges: Category I refers to High level - occupants with special needs (children, elderly, handicapped, etc.), Category II refers to Medium level, Category III to Moderate, and Category IV to Low - level that does not cause health risks, but reduces the comfort of users.

## References

[1] MACIEL Ana, Carolina F., CARVALHO Michele. (2019). Operational energy of opaque ventilated facades in Brazil. J. Build. Eng..

[2] RYU Han-Soo, PARK Kyuyng-Soon (2016). A study on the LEED energy simulation process using BIM. Sustainability.

[3] SANHUDO Luís, RAMOS Nuno M.M., MARTINS João Poças, ALMEIDA Ricardo M.S.F., BARREIRA Eva, SIMÕES M.Lurdes, CARDOSO Vítor (2018). Building information modeling for energy retrofitting – A review.

[4] IEA, International Energy Agency. Energy Technology Perspectives 2012. Pathways to a Clean Energy System (2012).

[5] Procel Info - Centro Brasileiro de Informação de Eficiência Energética Homepage, https://goo.gl/VpCYLd, last accessed 2017/12/10.

[6] SANJUAN Cristina, SUÁREZ María José, GONZÁLEZ Marcos, PISTONO Jorge, BLANCO Eduardo (2011). Energy performance of an open-joint ventilated façade compared with a conventional sealed cavity façade. Sol. Energy.

[7] ASTORQUI Jaime Santa Cruz, PORRAS-AMORES César (2017). Ventilated façade with double chambre and flow control device. Energy Build..

[8] ILBAÑEZ-PUY M., VIDAURRE-ARBIZU M., SACRISTÁN-FERNÁNDEZ J.A., MARTÍN-GÓMEZ C (2017). Opaque Ventilated Fcçades: thermal and energy performance review.

[9] MOON Hyeun Jun, SEOK Choi Min, KIM Sa Kyum, RYU Seung Ho (14-16 november 2011). Case studies for the evaluation of interoperability between a bim based architectural model and building performance analysis programs. Proceedinf of building simulation. Proceedings of the 12th Conference of International Building Performance Simulation Association.

[10] PAN Wei, QIN Hao, ZHAO Yisong (2017).

[11] KOTA Sandeep, STIPO Francisco J.Farias, JEONG Woon Seogn, KIM Jong Bum, ALCOCER Jose Luis Bermudez, CLAYTON Mark J., HABERL Jeff S (2016). Development of a reference building information model for thermal model compliance testing – part i: guidelines for generating thermal model input files. ASHRAE Trans..

[12] MAILE, T.; FISCHER, M.; BAZJANAC, V. Building energy performance simulation tools — a life-cycle and interoperable perspective, CIFE Working Paper #WP107, Stanford University (2007).

[13] DONG B., LAM K.P., HUANG Y.C., DOBBS G.M. (2007). A comparative study of the IFC and gbXML informational infrastructure for data exchange in computational design support environments. Proceedings of the Building Simulation 2007: Tenth International IBPSA Conference.

[14] KIM Jong Bum, JEONG Woon Seong, CLAYTON Mark J., YAN Jeff S.Harbel, Wei (2015). Developing a physical BIM library for building thermal energy simulation. Automation in Construction.

[15] U.S. Department of Energy (U.S. DOE), Building Energy Software Tools Directory Retrieved, http://www.energytoolsdirectory.gov (2012).

[16] PARK Jaehyun, PARK Junglo, KIM Ju Hyung, KIM Jaejun (2012). Building information modelling based energy performance assessment system: an assessment of the Energy Performance Index in Korea. Construct. Innov..

[17] SHOUBI Mojtaba Valinejad, SHOUBI Masoud Valinejad, BAGCHI Ashutosh, BAROUGH Azin Shakiba (2015). Reducing the operational energy demand in buildings using building information modeling tools and sustainability approaches. Ain Shams Eng. J..

[18] LU Yujie, WU Zhilei, CHANG Ruidong, LI Yongkui (2017). Building Information Modeling (BIM) for green buildings: A critical review and future directions. Autom. Constr..

[19] AZHAR Salman;, BROWN Justin. (2009). BIM for sustainability analyses. Int. J. Constr. Educ. Res..

[20] AZHAR Salman, CARLTON Wade A., OLSEN Darren, AHMAD Irtisha (2011). Building information modeling for sustainable design and LEED® rating analysis. Autom. Constr..

[21] BIM forum, BIM tools matrix, http://bimforum.org/wp-content/uploads/2011/02/BIM_Tools_Matrix.pdf, (2011).

[22] Autodesk, Autodesk building performance analysis help. http://help.autodesk.com/view/BUILDING_PERFORMANCE_ANALYSIS/ENU/?guid=GUID-43DAB177-3A4F-496C-BECB-2591FD04FC10, (2015).

[23] I. E. Solutions, Introducing IESVE Software, http://www.iesve.com/software, (2014).

[24] CBIC - CÂMARA BRASILEIRA DA INDÚSTRIA DA CONSTRUÇÃO. CUB/m²: Custo Unitário Básico. Indicador dos custos do setor da Construção Civil. Disponível em: < http://www.cub.org.br/cub-m2-brasil>. Acesso em 05 de julho de 2017.

[25] SINDICATO DA INDÚSTRIA DA CONSTRUÇÃO CIVIL NO ESTADO DE MINAS GERAIS (2007). Custo Unitário Básico (CUB/m²): principais aspectos.

[26] JUNGINGER M. (2003). Rejuntamento de revestimentos cerâmicos: influência das juntas de assentamento na estabilidade de painéis. Dissertaçao de Mestrado apresentado ao Programa de Pós-Graduação em Engenharia Civil.

[27] ASSOCIAÇÃO BRASILEIRA DE NORMAS TÉCNICAS. NBR 15575-1. Edifícios Habitacionais – Desempenho – Parte 1: Requisitos Gerais. ABNT, 2013. 83 p.

[28] ASSOCIAÇÃO BRASILEIRA DE NORMAS TÉCNICAS (2005). NBR 15220-3. Desempenho Térmico de Edificações - Parte 3: Zoneamento bioclimático brasileiro e diretrizes construtivas para habitações unifamiliares de interesse social.

[29] AMERICAN NATIONAL STANDARDS INSTITUTE. ASHRAE STANDARD 90-1: Energy Standard for Buildings Except Low-Rise Residential Buildings. Atlanta, 2013.

[30] KOTTEK M., GRIESER J., BECK C., RUDOLF B., RUBEL F. (2006). World Map of the Köppen-Geiger climate classification updated. Meteorol. Z..

[31] INTERNATIONAL STANDARD. ISO 17772. Energy performance of buildings – Indoor environmental quality – Part 1: Indoor environmental input parameters for the design and assessment of energy performance of buildings. ISO copyright office. Geneva, Switzerland, 2017.

[32] LEVIN Jack, FOX James Alan, FORDE David R (2012). Estatística para ciências humanas.

